# In/Fertile Monsters: The Emancipatory Significance of Representations of Women on Infertility Reality TV

**DOI:** 10.1007/s10912-019-09555-z

**Published:** 2019-04-06

**Authors:** Marjolein Lotte de Boer, Cristina Archetti, Kari Nyheim Solbraekke

**Affiliations:** 1 Department of Culture Studies, School of Humanities & Digital Sciences, Tilburg University, Tilburg, The Netherlands; 2 Department of Media and Communication, Faculty of Humanities, University of Oslo, Oslo, Norway; 3 Institute of Health and Society, Department of Health Sciences, Faculty of Medicine, University of Oslo, Oslo, Norway

**Keywords:** Infertility treatment, Infertile women, Reality TV, Monster, Content analysis

## Abstract

Reality TV is immensely popular, and various shows in this media genre involve a storyline of infertility and infertility treatment. Feminists argue that normative and constructed realities about infertility and infertility treatment, like those in reality TV, are central to the emancipation of women. Such realities are able to steer viewers' perceptions of the world. This article examines the emancipatory significance of representations of women on 'infertility reality TV shows'. While the women in these shows all have 'abnormal' qualities, we consider their portrayal as figurations of monstrosity. In the literature, monstrosity is understood as a way to challenge nonemancipatory norms by offering an alternative identity. Through a content analysis of seven reality TV shows, we identified four types of in/fertile monsters: the cyborg, the freak, the abject, and the childless. We show that these monsters are predominantly non-emancipatory as they all involve mechanisms of altering, excluding, or condemning infertility in relation to what is considered normal and acceptable womanhood. Therefore, at the end of this article, we make a plea for more diverse and emancipatory representations of infertile women in popular culture.

## Introduction

Over the past two decades, reality TV has increased immensely in popularity and become a fixture of television programming (Mast 2016; Murray and Ouellette 2004). Global television channels like MTV (Music Television) and TLC (The Learning Channel) even retooled themselves to focus predominantly on such programs.^1^ While reality TV broaches many topics, it saves a special place for women’s experiences of infertility and infertility treatment. In these shows, infertility struggles are typically spectacularized by featuring women with unusual lives and bodies. Such shows portray the extravagant lives of celebrities and their infertility issues (*Guiliana & Bill*; *Keeping Up with the Kardashians*), the struggles of a couple with dwarfism who try to conceive (*The Little Couple*), and all-American families that are completely normal except for the fact that they have multuplets as a result of infertility treatment (*Rattled*; *Jon & Kate Plus 8)*. Note that while the titles of most of what we call, “infertility reality TV shows” suggest that both partners are the main characters, they predominantly feature the women as the protagonists. This article analyzes the representations of these women on such reality TV shows.

Understanding the representations of women on infertility reality shows is important for two interrelated reasons: their potentially powerful influence and their emancipatory significance. Through their specific design, reality shows have a significant impact on the ways in which people come to understand and assess infertility and infertility treatment. Reality TV is often framed as entertainment and as representing the real experiences of real people. To underline this, the TV channel TLC previously used the slogan “Life Unscripted.” A large portion of reality TV, however, is explicitly formatted and namely for pedagogical purposes (Mast 2016; Sadowski-Smith 2014; Turner 2006; Tremlett 2014; Tully and Tuwei 2016). These shows are filmed and edited so that the viewers witness the participants’ struggles with complicated moral dilemmas (Sears and Godderis 2011). In doing so, the shows not only present the audience with the opportunity to judge the actions of those being watched, but they also steer viewers towards a certain kind of judgement. Reality TV thus offers a finely tuned education in governmentality cloaked with a façade of authentic and entertaining experiences that works to condition viewers to either make better choices than those depicted on-screen or to see the characters as role models in good decision-making (Ouellette 2014).

This constitutive role of reality TV is amplified by the fact that the issues presented in infertility reality shows are to some extent still shrouded in taboo and not openly debated. While in the Western world, the use of infertility treatments such as In Vitro Fertalization (IVF) seems to be a widely accepted option, other issues surrounding infertility such as childlessness, surrogacy as well as the emotional and physical burden fertility treatments places on women and couples is still censured and subject to disapproval (Michelle 2007). What the viewer learns on the screen about these issues could easily become all they know about the matter. In this way, screen content of infertility reality TV is not just a *representation* of reality; it contributes to the very construction of the reality the viewers live in.

Such normative constructed realities about infertility and infertility treatment are considered central to the emancipation of women (Franklin 1990; Thompson 2005). Feminists call on us to recognize that contemporary understandings of infertility and infertility treatment lend implicit support to biological kinship arrangements and to conventional and reductive gender roles, that is, to understanding the woman as a (future) mother, a caretaker, a walking womb, or living up to (very) high body standards. Several authors argue that within and through such understandings, a harmful culture is put into place in which women are made responsible for couples’ infertility; in which they are unhesitantly subjected to invasive medical fertility treatments; and in which undergoing such treatments appears effortless, or at least bearable (Thompson 2005; Strif 2005). In this context, then, emancipation would refer to a process of freeing women from these restrictive norms related to infertility. It would involve developing and accepting a frame of reference in which women are seen as independent of a hegemonic medical practice of infertility treatment and of essentialistic and often suppressive understandings of women (Yuval-Davis 1997).

The question of what kind of frame of reference reality TV offers regarding infertility is subject to debate. Several theorists celebrate reality series by arguing that, with the cameras left to roll on daily life, this TV genre provides some of popular culture’s more complex and comprehensive and therefore potentially emancipatory portrayals of lived experiences. Within this argument, infertility reality TV is assumed to show alternative and otherwise obscured choices, experiences, and lifestyles in relation to the status quo such as adoption, childlessness, male infertility, or couples’ shared suffering (Edge 2014; Murray and Ouellette 2004; Osborne-Thompson 2014). At the same time, in adhering to the fact that reality TV shows are still a popularized version of reality, scholars claim that such shows persistently traffic in conventional norms and sexist and ableist stereotypes while propagandizing unrealizable goals, life choices, and body standards (Ouellette 2014; Osborne-Thompson 2014).

While the existing debate about the emancipatory significance of reality TV shows offers interesting perspectives, its arguments and insights seem to be largely theoretically driven and lack systematic empirical grounding. The debate’s theoretical nature, moreover, seems to fuel its inherent polemics, namely where scholars argue either for or against reality TV’s emancipatory potential. In providing a better understanding of reality TV’s emancipatory significance, we aim to turn the current theoretical polemic about this television genre into a more complex and empirically grounded discussion. To this purpose, we offer an analysis of representations of women on infertility reality TV and think through their emancipatory significance. How are women who deal with infertility issues represented on reality shows? Do they break free of restrictive norms related to infertility? How or how not? What kind of repercussions could these representations have for the ways in which viewers come to assess and evaluate infertility and infertility treatment?

By drawing on hundreds of hours of footage from seven infertility reality TV shows, we will show that these shows represent perspectives on infertility that divert from the status quo: they show the bodily invasiveness and riskiness of infertility treatment, make the messiness involved in such treatments audible, and hint at the possible outcome of remaining childlessness. Such representations, however, are generally condemned, highly obscured, and subject to alteration. That is, women in infertility reality shows are predominantly displayed as willing and able to endure infertility treatment, to control and discipline their messiness and riskiness, and as eventually always becoming mothers. These women, in other words, are mainly represented as mothers or mother-to-be and as having disciplined, clean, and reproductive bodies. At the end of this paper, then, we will sketch out the possible harmful effects of these non-emancipatory representations on the ways in which viewers come to assess and evaluate infertility treatment. Before we turn to this empirical discussion, however, let us first elaborate on the theoretical framework that we use to interpret infertility reality shows.

## In/fertile monsters: from condemnation to liberation?

A specific theme running through reality TV shows about infertility is that the women depicted in these shows all have “abnormal” or monstrous qualities. They not only deal with the nonstandard issue of infertility but they often do so while living an extravagant life style, or in combination with physical anomalies, or while their stories culminate in excessive births. Considering the portrayal of these women as figurations of monstrosity helps to uncover and rethink their relations with the standards of normality and their emancipatory significance.

The image of the monster has a long history in popular culture, from Cyclopes in ancient Greek mythology, via scientific Frankensteinian creations, to the so-called freak shows at carnivals in the nineteenth century, and from bloody, slimy creatures in horror stories to the technology-human hybrids or cyborg figures in science fiction tales. While monsters show themselves in historically and culturally specific ways in various traditions, what they have in common is that they fall outside of the range of what is culturally regarded as a standard or ideal form of embodiment (Shildrick 2000, 2002).

Throughout history, the monster and its different embodiments have been regarded as having epistemological value. In medical research, monsters are seen as objects of knowledge and opportunities for the field to advance (Garland-Thomson 1997; Shildrick 2002). There is a rich medical tradition of researching humans with physical anomalies such as conjoined twins or humans without limbs (Daston 1991; van Dijck 2002). However, what remains unexplained about this epistemological fascination with the monster is that this figure also persistently invokes anxiety and fear in those who are confronted by it. To understand this, we have to turn to the political and ontological meaning of monster.

Many scholars assert that the image of the monster has long functioned (and still functions) as a scapegoat (Shildrick 2002; Haraway 1991; Braidotti 1994). The underlying logic for such scapegoating is that what is regarded as different must be located outside of the boundaries of what is perceived as normal: people of different races, foreigners, animals, disabled people, and women (Shildrick 2000, 2002). By way of their aberrant characteristics, monsters differ from a normative identity through which they become in some way blemished: improper, flawed, smeared, or soiled (Haraway 1991). In turn, the self is defined as normal or proper in relation to what is abnormal and improper. The monster thus signifies a politics of identity and difference: it carries a taint that must be excluded in order to define proper selfhood in relation to otherness and thereby secures a normative order.

However, as the French philosopher Jacques Derrida (1981) points out, such a politics of exclusion and of clear-cut boundaries is always an impossible one. Within and through defining otherness, he argues, selfhood is marked by the trace of this excluded other. After all, it is upon the exclusion of the other as different that the self as a proper subject relies. According to Derrida, therefore, otherness is to be understood at the heart of the identity of the self: every identity is always already worked upon by its own exteriority, by what is different (1981). Applying these workings of the self’s ambiguous ontology to the figure of the monster means that the monster has a constituting significance for the self, whereby the self necessarily keeps within itself a trace of monstrosity. As such, monstrosity is not only abhorrent exteriority, it is also enticing, a figure that calls to the self in a way that invites recognition (Shildrick 2000, 2002). It is through this play of monstrosity and the identification of otherness in selfhood, which threatens a clear-cut normative order, that this figure invokes fear and anxiety.

While monstrosity has long been a condemned category, theorists such as Michael Bakhtin (1984), Donna Haraway (1991), Rosi Braidotti (1994), Mary Russo (1995), and Margrit Shildrick (2000, 2002) have made it a category that pushes a more positive, feminist agenda. “Any being,” Shildrick writes, “who traverses the liminal spaces that evade classification takes on the potential to confound normative identity, and monsters paradigmatically fulfill that role” (2002, 5). As the monstrous other is always identified within ourselves, being confronted with this figure provokes us in our thinking not simply about the binary encounter between the self and the other but about the very impossibility of such a dichotomous position. Thus, by adhering to our basic ontological structure, monsters create openings for thinking through normative identities and systems of exclusion and can be understood as having emancipatory significance.

Looping back to the topic of infertility reality TV, we may ask whether this argument about the emancipatory significance of monsters can be expanded to representations of in/fertile women as monsters. As the monster’s subversiveness is ambiguous—it questions and breaks the norm but at the same time constructs and reinforces it—the question arises whether the representation of women as in/fertile monsters underscores the often restrictive norms related to women’s infertility, or whether these monsters challenge such norms. Whether and how does the portrayal of women on infertility reality TV confront us with abnormal, strange, undesirable, or even abhorrent bodies and selves in ways that question the status quo?

## Researching fertility reality TV

Because this study aims to analyze the emancipatory significance of representations of women on infertility reality TV, we have chosen to conduct a content analysis that identifies and analyzes recurring themes and storylines related to non/emancipatory portrayals of women undergoing fertility treatment. In this study, we included excerpts of American, English-speaking reality television shows (scenes, episodes, and/or whole seasons) and related video content (personal YouTube vlogs of the main characters, so-called online “teasers,” and follow-up TV episodes after the actual reality series has ended) that incorporate infertility and infertility treatment as one of the storylines. This means that reality TV shows were selected that deal with infertility and infertility treatment in at least two of their episodes and/or related video content. This resulted in the inclusion of seven reality TV shows and hundreds of hours of visual material in the analysis.

The included shows *A Baby Story* and *A Conception Story* deal with having a baby and conceiving in general, but they also feature several couples who elaborate in front of the camera about their struggles with infertility. These shows alternate between interviews and peeks into the cast members’ daily lives like going to the fertility clinic or doing household chores. The shows *The Little Couple*, *Giuliana & Bill* and *Keeping Up with the Kardashians* also focus on couples’ infertility struggles, but they do so within the overall story of the newlywed life of a couple with dwarfism (*The Little Couple*), a couple’s life of stardom (*Giuliana & Bill),* and a family’s life of stardom (*Keeping Up with the Kardashians)*. The series *Jon and Kate Plus 8* and *Rattled* feature infertility storylines within the larger narratives of families with multuplets as a result of infertility treatment. See Table 1 for more information about the shows.

**Table 1:** Information about the analyzed infertility reality shows

Shows	Characters and depicted infertility treatments	Included video content
*A Baby Story* (Discovery channel and TLC, 1998 – 2010)^a^	Storylines of:	
	1. Trish and Andrew: IVF treatment, expecting a baby. 2. Stephanie and John: IVF treatment, expecting a baby 3. Christina and Ron: IVF treatment, expecting a baby.	Season 2009, episode 8 (Trish and Andrew) Season 2009, episode 3 (Stephanie and John) Season 2010, episode 11 (Christina and Ron)
*A Conception Story* (TLC web series, 2010 – 2012)	Storylines of:	- The short online videos of season 1 and 2, and several personal YouTube vlogs in which the couples Becky and Steve, Teresa and Jason, and Bari and Chad are featured.
	1. Becky and Steve: IVF treatment, expecting a baby	
	2. Teresa and Jason: IVF treatment, not able to conceive, discussing the option to adopt.	- Follow-up YouTube videos featuring Becky and Steve, and Bari and Chad.
	3. Bari and Chad: IVF treatment, expecting a baby	
*Guiliana&Bill* (Style Network and E!, 2009 – 2014)	Stardom couple Guiliana Rancic (television presenter) and Bill Rancic (entrepreneur).	Season 2, episodes 1 -8
		Season 3, episodes 1 - 10
		Season 4, episodes 1 - 10
		Season 5, episodes 1 – 21
	Several IVF treatments, eventually opting for gestational carriership which results in having a baby.	‘Teaser’-videos on Youtube.
*Kate and Jon plus Eight* (TLC, 2007 – now as ‘*Kate plus eight*’)	Couple Kate and Jon, parents of twins and of sextuplets, all of which are the result infertility treatment.	Season 1, episode 1 and 2. ‘Teaser’-videos on Youtube.
*Keeping up with the Kardashians* (E!, 2007 - now)	Stardom family Kardashians, and specifically the sisters Chloé and Kim Kardashian, who both deal with infertility. Sister Chloé offers to be the gestational carrier for Kim.	Season 13, episode 13 and 14.
*Rattled* (TLC, 2016 – now)	Couple Tyson and Ashley, parents of quadruplets, which are the result of infertility treatment.	Season 1, episodes 1 – 6
		Personal vlogs on their family YouTube channel ‘Gardner Quad Squad’.
*The Little Couple* (TLC, 2009 - 2016)	Couple Bill and Jenn, who both have dwarfism. Several rounds of unsuccessful gestational carriership. They eventually adopt two children.	Season 1, episode 14,
		Season 2, episodes 1, 15 - 17
		Season 3, episodes 1 - 16
		Season 4, episodes 1 - 24
		Season 5, episodes 1 - 35

^a^Authors were only able to include the episodes of this show after 2008 because the older episodes were not available anymore

The first author watched and re-watched the selected fertility reality shows and identified recurring themes and storylines related to non/emancipatory portrayals of women in infertility treatment. The most prevalent themes identified in these reality shows were: ‘women as only mothers or mothers-to-be’; ‘women should (be able to) conceive (naturally)’; ‘women being made responsible for in/fertility issues and choices’; ‘women being blamed for in/fertility issues and choices’; ‘women disciplining themselves and their bodies during infertility treatment’; ‘women with perfect or perfected bodies.’

In consultation with the other authors of the article, this analysis resulted in the identification of four types of representations of in/fertile women as monsters, namely as cyborgs, as freaks, as abject, and as childless. The cyborg and the freak are explicitly and extensively represented in reality shows, while the abject and the childless are more obscure in their representations, that is, merely represented in sound (abject), or as suggested in its absence (childless). All of these monsters incorporate one or more renditions of the identified themes related to women’s non/emancipatory portrayals.

## The cyborg: unnatural conception and natural mothers

Within all the analyzed shows, the female protagonists engage extensively with a wide range of medical technologies, tools, and artifacts. The series follow women into the consultation room of the fertility clinic and show how they engage with medical machinery and equipment such as ultrasound wands, specula, surgical knives, tubes, monitors, and microscopes. Reality shows also depict how medical artifacts like ovulation, pregnancy tests, thermometers, needles, and pills are extensively used at home. In reflective interludes, several women talk in front of the camera about how their bodies react to and change with the usage and incorporation of these technologies and artifacts. Given these elaborate portrayals of women engaging with various kinds of infertility technologies and artifacts, the cyborg stands out as a significant version of the represented in/fertile woman. That is, women are represented as human-technology hybrids in the sense that their bodies are neither depicted as being completely determined by their human organic physicality nor only by the usage and integration of the artificial and technological artifacts but by a complex combination thereof (Haraway 1991).

A rich tradition of feminist literature celebrates such cyborgs figures for disrupting persistent dualistic understandings of the self and the body as either natural and material-biological or as culturally and technologically crafted (Haraway 1991; Balsamo 1996; Lykke and Braidotti 1996). Such dualisms have been systemic to the logics and practices of the domination and suppression of women. Throughout history, different configurations of the appropriation of nature figure as a resource for the productions of (normative) culture. Such configurations state, for example, that nature designed women to be nurturing in that they are able to become pregnant and breast feed, and so they are supposed to stay home with the children. Cyborg bodies challenge such thinking in that they make thoroughly ambiguous the difference between natural/organic and cultural/technological because of the indeterminacy of their hybrid design. In her famous manifesto, Haraway states that in a cyborg, “it is not clear who makes and who is made, (and as such,) there is no fundamental, ontological separation in our formal knowledge of machine and organism, of technical and organic” (1991, 177–178). Nature and culture, then, are understood to be enmeshed in the cyborg, and thus one cannot be used as the resource for appropriation or incorporation by the other.

Some of the reality series’ narratives resonate with this argument in that the depicted women seem to experience being (and becoming) cyborgs as disruptive to their understanding of themselves and their bodies. While some women simply refer to the “foreignness” (Giuliana) or “weirdness” (Becky) of using technologies and machinery in their fertility treatment, Ashley, from the show *Rattled*, gives a more explicit and elaborate account of how fertility technologies disrupt and change her understanding of her body. She says:

You just think that you are able to become pregnant naturally, (…) and you don’t. Now I need a doctor and all this machinery and that’s just… different… (…) He [the fertility doctor] is their [her babies’] maker now, not us. (…) Or well, “maker,” that’s maybe too much, [but] he had a lot to do with it, but us too ha ha.

In reflecting on her infertility treatment experiences, Ashley hints at understanding her body while it is undergoing infertility treatment along dualistic lines. She begins referring to a “natural” pregnant body, one that she thought she would have but has not: a body that is able to conceive without technological assistance. In describing becoming pregnant through fertility treatment, she initially designates the fertility doctor as the “maker” of her children, thereby implicitly referring to an understanding of her pregnant body, and that of her babies, as merely technologically crafted. Immediately, however, she places reservations on this understanding, saying that both the doctor and they as a couple “had a lot to do” with her becoming pregnant. While Ashley seems to start out with a dualistic and mutually exclusive understanding of her body as not “natural” and therefore “technical,” she ends up with a less rigid and dichotomous, and perhaps more complex, bodily understanding.

Note that for many women, the experience of using and needing to use fertility technology in order to have a baby is a difficult one. Just like Ashley, they have to adjust their expectations of being able to conceive a baby “naturally,” if ever. Just as some women state they feel they are supposed to conceive “naturally,” they also have to give up the idea of living up to this norm. Stephanie from *A Baby Story* narrates this by saying:

Going to a doctor’s office to have a baby is something that I had to come to terms with. You know, I always saw myself remembering the intimacy when we conceived our baby. [But also,] it feels like I failed myself and John [her husband] for not being able to get pregnant naturally.

For most of the women in the shows, however, infertility treatment is generally the only option to have a (biological) baby. As a result, they work, in the words of Stephanie, on “coming to terms with” needing and using fertility treatment. Many of them do so by familiarizing and adjusting themselves and their bodies to the infertility treatments, technologies, and tools. Giuliana from *Giuliana & Bill*, for example, reads up on what the various infertility treatments entail and how the technologies and tools work, thereby, as she says, “making them less weird. [Now,] I can see myself doing it.” Ashley’s way of adjusting to the fertility technology is an explicitly embodied one. In recounting the experience of having intra-uteral surgery as part of her treatment, Ashley says:

It’s incredible what they can do with the technology. But of course, I had to prepare for it as well: resting, being in shape, and so forth. That was hard, especially resting—then my mind kept spinning…what if?! But well, it [the treatment] worked immediately and that’s rare.

Both Giuliana and Ashley work on making the fertility treatments and technologies fit their actual bodies or their ideas about themselves. In doing so, these women not only familiarize with and adjust to the technologies at hand, but they also try to make their bodies and lives more welcoming and even receptive to such technologies (Winance 2010). It is within and through such work, then, that these women can be understood as becoming cyborgs.

Given that many women on reality shows have to go through an intensive and difficult process of redefining themselves in relation to the “weird,” “foreign,” or “different” fertility tools and technologies, it is surprising that opting for using fertility technologies is also described as “a normal way to go [in conceiving]” (Bari/*A Conception Story*). This multifaceted perception of fertility technologies as being both “weird” or “different” and “normal,” seems to be, on the one hand, caused by the disruption of these women’s normative views of their reproductive bodies as natural. On the other hand, such technologies seem to be regarded by many women as also normal because they implicate a horizon of living up to another kind of norm, that is, the social obligation of becoming a mother (Balsamo 1996). Bari illustrates this nicely when she explains why for her, infertility treatment was “a normal way to go”: “Like all girls, I always wanted to become a mother…I am supposed to be one. It’s just in our DNA.” Bari’s statement implies that fertility technologies can be seen as a means of correcting bodily insufficiency in order to live up to the standard or norm that women have children. In living up to this norm, Bari even appeals to her “natural,” or biologically deterministic purpose: being a mother. Interestingly, this shift from being estranged from, to welcoming, to normalizing infertility treatments is also reflected in and underscored by the ways in which the infertility shows are edited. Both Giuiliana’s and Ashley’s infertility story is initially accompanied with dramatic visuals of suddenly zooming into infertility technologies and artifacts. When their story develops towards integrating these technologies into their perception of themselves, however, the camera refrains from isolating these artifacts but rather shows the women in relation to them: as being attached to some apparatus or as putting needles into their bodies. In Ashley’s case, moreover, these relational human-technology visuals are predominantly used as glimpses into her (infertility treatment) past and are often alternated with footage from the children that she had as a result of her infertility treatment. In this way, then, it may be interpreted that Ashley’s welcoming attitude towards fertility treatments is visually depicted as paying off in enabling her to live up to the norm of having children. These visuals, then, as well as the women’s linguistic stories reveal that becoming a cyborg within and through infertility treatment is a paradoxical position: while it may disrupt women’s understanding of themselves and their bodies as “natural,” it also enables them to live up to their supposedly “normative” and “natural” purpose in life. So rather than that the cyborg’s hybridity is emancipator—as is assumed in the literature—it seems to be a way to accommodate and support a non-emancipatory cultural appropriation of nature, namely where women are perceived as “natural” mothers or mothers-to-be.

## The freak: disciplining and blaming risky women

While all infertility reality TV series feature cyborgs, some also depict women with freakish or carnivalesque qualities, namely women who are pregnant with multuplets as a result of infertility treatment (*Rattled; Kate & Jon Plus 8; A Baby Story*), and a little person who tries to become pregnant through surrogacy and infertility treatments (*The Little Couple*). The figure of the freak—which harkens back to the tradition of freak shows and carnivals—is commonly understood as hyperbolically representing otherness, usually by way of excessive or inversive characteristics (Harpham 1982). In this case, the excessively pregnant women or the unusually little person do not fit into the standard and normative categories of selves and bodies.

Several social theorists point out that the display of the freak confronts us with the difficulty of naming the boundary between culturally defined otherness and selfhood (Bakhtin 1984; Derrida 1981; Stewart 1993; Russo1995). The freak not only affirms the self as self in relation to freakish improperness, but the freak is also doubly marked as the tamed and bewildering other. By drawing on historical data about freak shows where freaks were often caged, silenced, and presented as animal-like, Stewart argues that this figure is “domesticated in a process we might consider to be characteristic of colonialization in general. (…) The freak represents the assurance that the wilderness, the outside, is now territory” (1993, 109–110). Bakhtin (1984) argues that freakish bodies not only represent the power of taming but, by displaying their unusual characteristics and unruly corporeal qualities, they also invite imaginations of otherness –bodily otherness, but also social and cultural differences. The freak, he claims, “discloses the potentiality of an entirely different world, of another order, another way of life" (Bakhtin 1984, 48). It is in this sense that, for Bakhtin, freaks both indicate and serve as a challenge to the status quo, even as a kind of visual road map to resistance, dissent, and bewilderment (Russo 1995).

At first glance, however, the freakish women on reality shows are not surrounded by discourses and practices of rebellion but rather by that of disciplining, regularization, and control. Although the story of a little person seeking infertility treatment and that of women carrying multuplets seem vastly different from one another, what these narratives have in common is that these women and their choices, lifestyles, and bodies are presented as being at risk. Jenn from *The Little Couple* explains that for her as a petite little person, becoming pregnant means risking her life. While seeking gestational carriership, surrogacy, or adoption as an alternative, Jenn and her husband Bill are visited by a social worker who determines whether their house, which is tailored to their height, is suitable and not dangerous for children. In the shows *Rattled* and *Jon and Kate Plus 8*, moreover, the voice-over constantly reminds us that for Ashley and Kate, carrying multuplets is risky for both them and their babies’ physical wellbeing. Besides narrating these women’s post-infertility treatment lives as a health risk, the show also depicts their lives as a financial and organizational risk because of the fact that they have to take care of multuplets. An interview scene with Kate from *Jon and Kate Plus 8* illustrates this:

Interviewer: “Do you feel like you did the right thing now that you know you have sextuplets?”

Kate: “Well, the goal of infertility treatment is to get one. The chance of having sextuplets is slim. But did I know the risk? Yes. (…) Was it smart? Perhaps not. I mean, we could hardly manage with two children, bringing them to all their clubs and stuff. (…) And we could not even afford the two we already had, let alone six more.”

While such narratives of risk, as Russo (1995) explains, point to taking a chance—in this case, to allow for the possibility of having a child through infertility treatment—it also refers to “errors” or “surprises.” Taking a risk, then, is the “possibility [of either] liberation and happiness [or] misery, injury and even death” (1995, 13). The logic of modern life, however, aims to dissociate itself from mistakes or surprises, namely through models of proven workability, sustained progress, and rationality (Beck 1992). Women’s presented risky choices, bodies, and lifestyles on reality TV shows are therefore accompanied with practices of disciplining—monitoring, control, and normalization—in order to decrease or ease the possibility for surprises and mistakes. During their pregnancies with multuplets, both Kate and Ashley are put on strict hospital bedrest for months because, as Kate explains, “the doctors were scared and protective: they have never dealt with a person like me before.” Ashley, in one of her YouTube videos, reacts to her fans checking whether she indeed stays in bed: “Rest assured,” she says, “I am only leaving this bed to go to the bathroom.” Jenn is advised by her doctor not to take the risk of becoming pregnant. Opting for surrogacy, gestational carriership, or adoption, is, as he says, “the safer alternative.” And the social worker who visits Jenn and Bill designates their house as having “red flags that could pose extreme dangers to a child.” With door handles in reach of toddlers, Bill and Jenn are advised to lock the doors in such ways that even they cannot open them. “It’s ironic,” Jenn remarks, “that what works well for us is the opposite for a child because we’re the same height as a toddler.”

Although all these women-freaks are subjected and subject themselves to disciplining practices, it turns out that surprises can never be fully avoided. Both Kate and Ashley refer to the unexpectedness of being pregnant with multuplets. Kate, for example, says she was “overthrown” by the “bombshell” of being pregnant with sextuplets. Now that she has eight children to raise, she characterizes her life as a “minefield of surprises.” Note that within such narratives of surprise, the women in the shows are generally framed as being responsible for unexpected events or surprises, especially when they turn out to be “bad” ones. In response, some of them plea bargain for failing to avoid surprises and to take full control. For instance, in a special interview episode, Kate publicly owns her “guilt” for having multuplets when she could not afford them and then publicly seeks redemption by apologizing for this “mistake.” After this interview, the remaining episodes of the show’s season focus less on the problematic side of raising eight children and more on its joyuous aspects, thereby suggesting that Kate is only able to control her life by owning up to her “mistake,” or even that she has been “forgiven” for this “mistake.”

Not all women, however, feel responsible or guilty for not being able to elicit successful risk control tactics. Jenn and her husband Bill, for example, challenge and negotiate with the social worker who declares their house risky. Jenn says, “We will make some changes, but we are not going to ‘pad’ the whole house. (…) We take the chance that it can go wrong. But that’s just life.” Bill goes on to say that “by not adjusting to average height life all the way, I hope to show the audience that even with our ‘abnormal’ lives, we are perfectly capable of raising a child. (…) Hell, our house might fit a toddler better than a ‘normal’ house.” Within and through such challenges and negotiations, “freaks” like Jenn and Bill show that, against traditional readings of these figures, they are not victimless and powerless (See also Russo 1995). It is thus particularly in *The Little Couple* that the double mark of the freak as both “tamed” and “bewildering” stands out. While all the infertility reality TV freaks in this article are willing to comply with norms related to being and becoming pregnant, to nuclear family arrangements, and to providing a child-proof home, Jenn and her husband Bill make a point that being a good parent may also involve not living up to the norm. In this sense, the representation of women as freaks on infertility reality TV is both underlining and resisting the status quo, and therefore, can be regarded as having non-emancipatory *and* emancipatory significance.

## The abject: disciplining appearances and perfected bodies

Besides from the cyborg and the freak, there is another, invisible but still present monster represented in infertility reality shows: the abject woman. In these shows, we do not see women bleed, leak, vomit, lactate, poop, or pee. Even if the narrative includes physical acts of labor, miscarriage, breast feeding, morning sickness, or complications during the treatment—bleeding, leaking, puking situations par excellence—we merely see women with clean, unblemished bodies and perfectly make-uped faces. On reality TV, women’s bodies are generally not visualized as abject, that is, as messy, sick, and damaged. Rather, these women are displayed as successfully disciplining their bodies to a state of constant control over themselves and their bodily fluids.

This lack of abject bodily visuals can be understood as carrying moral and social significance (Waskul and van der Riet 2002). This is because the abject refers to a realm that is impure, unclean and disorderly (Shildrick 2000, 2002; Kristeva 1982). By dispelling what is normally inside, the abject is a source of horror and repulsion, and therefore of disturbance, disorder, and even ominous seepage to spectators (Kristeva 1982). The abject, as Julia Kristeva aptly writes, is “what disturbs identity, system and order, [it is] that which does not respect boundaries, positions, rules” (1982, 4). By not visualizing abjectness and excluding footage that may be perceived as impure and unclean, reality TV seems to feed into the perception that women are able to discipline and perfect their bodies to proper ones during infertility treatment, thereby securing the order of the proper.

There are, however, other kinds of presentations of abjectness on infertility reality TV, namely in sound. During an egg retrieval procedure scene in *The Little Couple*, we hear sounds of flapping flesh, dripping fluids, and squishing slimes. Meanwhile, we are looking at the door of the room from which these sounds supposedly come. Interestingly, these audible representations of abjectness do not seem to be disruptive ones, at least not too much. They only seem to be acceptable ones (i.e. not *too* disruptive) because hearing abjectness is not as intense as both hearing and seeing, or as only seeing the abject. This is because audible abjectness is less likely to cause powerful reactions of disorder and disgust (Hanich 2009). Of course, taking away one of the sensuous stimuli on reality TV—either sight or hearing—already diminishes the intensity of the abject, but particularly by obscuring the abject, its disruptive power decreases. This becomes clear when we turn to the meaning of our senses in bringing forth powerful disruptive experiences of abjectness, like horror, repulsion and disgust.

The so-called immediate senses of touch, taste, and smell are generally regarded as the most horror and disgust-ridden senses because these reactions predominantly depend on the subject’s direct contact with its stimulus (Plantinga 2006; Miller 1997). To be disgusted means to feel the slimy and sticky, to smell the foul, or to taste the loathsome. However, the audio-visual medium of television, which relies on the alleged “distant” senses of hearing and sight, can also cause strong-lived and bodily responses typical of disgust even if the senses of touch, smell, and taste are not directly called upon. Here, disgust requires an act of imagination to put oneself into a condition of direct exposure to the disgusting object (Plantinga 2006). As the word suggests, imagination works with images, with visualizing something or someone. Visual shots can be appalling because we imagine ourselves touching, tasting or smelling the presented object. Compared to the other senses, then, hearing plays the most indirect and consequently least significant role in processing horror and disgust. Sounds only disgust or horrify through the detour of imagination and visualizing images of loathsome touches, horrible smells, or gagging tastes. Hearing abjectness is the least likely of all senses to process intense bodily reactions of disgust or horror. Thus, as audible representations of abjectness do not come across as *too* messy or *too* disruptive for the order of the proper, infertility reality TV keeps up appearances: by merely making improperness audible, it predominantly underscores the non-emancipatory view that women are able to, and should have, perfect, clean, and proper bodies during infertility treatment, or at least that they do not disrupt it too much.

## The childless: the ultimate provocation of womanhood

Compared to the abject, another kind of monster is even more absent on infertility reality TV: the childless woman. Infertility reality TV predominantly depicts women who (eventually) have children, namely in the aftermath of successful infertility treatment (i.e. *Jon and Kate Plus 8*, *Rattled*, *A Baby Story*) or during infertility treatments that turn out successful during the show (i.e. *Keeping Up with the Kardashians, Giuliana & Bill, A Conception Story)*. Furthermore, in the few instances in which an unsuccessful treatment trajectory is narrated, the shows follow the couples into the route of adopting a child (i.e. *A Conception Story, The Little Couple)*. On infertility reality shows, infertility struggle narratives are generally stretched out over multiple episodes and often function as cliffhangers in season finales. Having a baby is presented as the climax of overcoming couples’ struggles. Even more, having a baby is explicitly or implicitly depicted as diminishing or even overcoming one’s monstrosity. For instance, the voice-over in *Jon and Kate Plus 8* repeatedly states that although Jon and Kate’s household is “excessive,” “an exception,” or “unusual,” Kate is “also just a normal mother.” And when Jenn and Bill become parents, the opening title sequence of the show mainly introduces them as parents instead of predominantly focusing on their freakish height, as was the case in the pre-parenthood seasons. That is, in the pre-parenthood episodes, Jenn and Bill are depicted in the opening sequence as standing next to a random object (chair, house) and average height people, thereby emphasizing their abnormality. After becoming parents, however, such visuals have disappeared from the opening sequence, and Jenn and Bill are mainly introduced by showing clips of them interacting and taking care of their children.

Interestingly, in the one case in which a reality show’s narrative gears towards a couple remaining childless, its storyline is canceled from the show. Without reasoning and rather abruptly, the couple, Teresa and Jason, from *A Conception Story* do not return to television after the episode in which it becomes clear that their treatment is unsuccessful and that they are still debating the option to adopt. They are not even featured in a follow-up episode a year after the season ended, an episode in which all the other couples appear. Thus, while not explicitly represented, the presence of childless women is suggested on infertility reality TV namely by featuring their storyline up to the point that a childless horizon is foreshadowed, after which their story is cancelled from the show.

This kind of portrayal of childless women on infertility reality TV is, figuratively speaking, a snake that bites its own tail. By never explicitly depicting childlessness and only following women into motherhood, these shows affirm the normative promise of biomedicine to mold and shape women’s bodies so that they can conceive (Wagner 2000), while underscoring the continuing cultural discourse that mothering is essential to womanhood (Phoenix et al. 1991). However, as reality TV depends to a certain extent on the accidental unfolding of events during infertility treatment, it seems that the possible outcome of remaining childless despite such treatment cannot be denied entirely. Consequently, childlessness is suggested before the cancellation of a “remaining childless” storyline, and it is such an abrupt and radical cancellation that its disruptive power echoes. Childless women in the aftermath of infertility treatment, after all, would personify the failing of the biomedical promise and implicitly contest the persistent ideal of women as mothers (Phoenix et al. 1991). While obscuring childless women on infertility reality TV seems to secure these existing normative and arguably non-emancipatory frameworks of biomedicine and womanhood, the ways in which these women are radically obscured, and the supposed need thereof, challenge the security of these non-emancipatory norms. After all, obscuring childless women testifies to the fact that women are not always mothers or mothers-to-be.

## Conclusion and discussion

In this article, we have taken the figure of the monster as a theoretical tool in analyzing and discussing the emancipatory significance of women’s representations on infertility reality TV. Monstrosity has come to be seen as a way to question and challenge normative identities through offering an alternative. This monster’s subversiveness, however, is ambiguous: it breaks the norm but at the same time reinforces it. As such, the question arises whether the representation of women as in/fertile monsters is just subject to such reinforcement, or whether they may actually challenge the norm, and thereby, help an emancipatory process of freeing women from restrictive norms related to infertility.

Our analysis shows that infertility reality shows, within and through their rationale of spectacularizing women’s (alleged) infertility and their difficulties in conceiving “naturally,” all represent their female protagonists as monsters. In doing so, the narratives’ emphasis is on these women’s difficulties in understanding themselves as normal women in light of their infertility issues and as such enforces a restrictive understanding women’s identity as being dependent on their ability to “naturally” conceive and have a baby. This narrative of condemned monstrosity is underlined by the fact that all the women in these shows try to escape their tainted identity by opting for infertility treatment—and consequently becoming another kind of monster: a cyborg. In what seems to be a tradeoff, women settle for an “unnatural” conception through which they are able to live up to what they regard as their “natural” purpose in life: becoming mothers. This dynamic of normalizing infertile women’s diversion from the norm is also present in two other monsters. As freaks and as abject, women are depicted as able and willing to discipline themselves by controlling and standardizing their lives and bodies in infertility treatment, i.e. their precarious life styles and choices (freaks) or their messy bodies (abject). Then, if these monsters fail in disciplining their unruly selves, lives, and bodies, they take the blame (freaks) or are hidden from view (abject). Through such blame-taking and obscurity, then, these women seem to be able to take control (again) and discipline themselves and their bodies. Moreover, if these cyborg-women fail to live up to their promise of conceiving through technological assistance, they become entirely imperceptible, albeit their existence is suggested. Through such obscurement of childless women after infertility treatment, motherhood is not only deemed as an essential role for women, but also presented as a deterministic one because of the supposed infallibility of infertility treatments. All women on infertility reality TV at least *appear* to become mothers.

Although reality TV tackles some infertility struggles and hints at possible childlessness, representations of in/fertile monsters predominantly involve a mechanism of altering, excluding, and/or condemning infertility—and the riskiness, messiness, and possible childlessness that comes with it—in relation to what is considered normal and acceptable womanhood. While we cannot outline the actual effects of these distorted and limited representations of lived infertility on audiences, we can point out that such representations may have significant and potentially harmful repercussions for the ways in which viewers come to assess and evaluate infertility. After all, reality TV is argued not only to have a constructive but also an educational function in (steering) viewers’ perceptions of the world. When confronted with these non-emancipatory portrayals of in/fertile women, viewers construct and/or adjust their lived reality—and assessment thereof—accordingly. Viewers may then endorse perceptions that women’s essential role in life is motherhood, that not being able to conceive “naturally” is a matter of failing womanhood, that women are responsible and have to take the blame for their infertility and their reproductive choices, that they must be willing and able to discipline their unruly lives and bodies during infertility treatment, that they are always able to conceive with technological assistance, and ultimately, that remaining childless is not an acceptable option.

At the end of this paper, then, we would like to make a plea for more diverse representations of infertile women in popular culture. This may entail reproducing “abnormal” and “risky” bodies in infertility treatment not as part of a culture of blaming and shaming, but just as a part of life that should not necessarily be erased. Such representations may also involve actually showing abjectness in infertility treatment, that is: visualizing women giving birth, having miscarriages, or being sick. It may also include countering the current pro-natal representations by incorporating voices and experiences of childless women. The point of this more broadened representation is not only to negotiate, challenge, and transform the dominant cultural depiction of infertile women as abnormal, different, or as dirty, but—perhaps surprisingly—also to reproduce such perceptions, albeit while carrying a different meaning. Such representations, after all, could show that falling outside of what is regarded as normal—taking risks, messiness, and the possibility of ending up childless—may be part and parcel of experiences of infertility and infertility treatment and do not have to be subject to condemnation, alteration, or elimination. It is in this way that monstrous representations can be perceived as truly being emancipatory: in their representation, we would not only normalize abnormality—or abnormalize normality for that matter—but also acknowledge and appreciate abnormality in and for itself.
